# An Implanted Magnetic Microfluidic Pump for In Vivo Bone Remodeling Applications

**DOI:** 10.3390/mi11030300

**Published:** 2020-03-13

**Authors:** Ziyu Chen, Sunggi Noh, Rhonda D. Prisby, Jeong-Bong Lee

**Affiliations:** 1 Department of Electrical Engineering, The University of Texas at Dallas, Richardson, TX 75080, USA; z.chen@utdallas.edu; 2 Department of Kinesiology, The University of Texas at Arlington, Arlington, TX 76019, USA; sunggi.noh@mavs.uta.edu (S.N.); rhonda.prisby@uta.edu (R.D.P.)

**Keywords:** intramedullary cavity, microfluidic pump, magnetic, implantable

## Abstract

Modulations of fluid flow inside the bone intramedullary cavity has been found to stimulate bone cellular activities and augment bone growth. However, study on the efficacy of the fluid modulation has been limited to external syringe pumps connected to the bone intramedullary cavity through the skin tubing. We report an implantable magnetic microfluidic pump which is suitable for in vivo studies in rodents. A compact microfluidic pump (22 mm diameter, 5 mm in thickness) with NdFeB magnets was fabricated in polydimethylsiloxane (PDMS) using a set of stainless-steel molds. An external actuator with a larger magnet was used to wirelessly actuate the magnetic microfluidic pump. The characterization of the static pressure of the microfluidic pump as a function of size of magnets was assessed. The dynamic pressure of the pump was also characterized to estimate the output of the pump. The magnetic microfluidic pump was implanted into the back of a Fischer-344 rat and connected to the intramedullary cavity of the femur using a tube. On-demand wireless magnetic operation using an actuator outside of the body was found to induce pressure modulation of up to 38 mmHg inside the femoral intramedullary cavity of the rat.

## 1. Introduction

Osteoporosis is a bone disorder that increases a person’s risk of fracture due to low bone mineral density, impaired bone microarchitecture/mineralization, and/or decreased bone strength [1]. Osteoporosis has become a major public health issue in recent years, especially in people of old age. Approximately 10 million men and women in the United States have osteoporosis [2], leading to 1.5 million bone fractures per year in the U.S. alone [3]. It is known that increased physical activity augments bone mass because of bone mechanical loading, while reduced physical activity (e.g., sedentary lifestyle) diminishes bone mass [4,5]. To date, the exact cellular and molecular mechanism of bone remodeling initiated by mechanical loading remains largely unknown [6]. 

Evidence accumulated over the years has suggested that one of the primary causes of bone remodeling is due to intramedullary fluid flow fluctuation under mechanical loading [7,8]. Previous in vitro studies have suggested that fluid flow inside the bone intramedullary cavity regulates bone cellular responses in bone cells (i.e., osteoblasts, osteoclasts, and osteocytes) that enhances bone formation and/or inhibits bone resorption [9,10]. It is suggested that shear stress induced by augmented fluid flow and pressure in the cavity elicits the release of bone stimulating factors [11], such as nitric oxide (NO) and prostaglandins (e.g., PGE_2_) [12,13]. Nitric oxide inhibits osteoclastic degradation of bone [14], while PGE_2_ enhances bone formation and reduces bone loss with immobilization [15]. Static loading, on the other hand, has little effect on fluid flow and does not elicit the same responses in bone cellular activities [9,16]. In contrast to the amount of studies performed in vitro, in vivo studies that demonstrate modulated intramedullary fluid pressure and/or flow mediate bone remodeling in response to mechanical loading are scarce. In vivo studies of modulated mechanical loading have been reported to mitigate bone loss and enhance bone formation in various animal models [17,18,19,20,21,22]. A few studies have also demonstrated that oscillatory intramedullary fluid flow loading alone in the absence of bone mechanical loading can result in the desired augmentation of bone formation in turkey ulnae [23] and hindlimb suspended mice [24]. These observations suggest that intramedullary fluid flow and pressure modulation without mechanical loading could be a non-pharmaceutical alternative to treat osteoporosis and facilitate bone fracture healing.

Despite its great potential value, in vivo studies altering bone intramedullary fluid pressure/flow without mechanical loading and its efficacy to initiate bone remodeling have not been fully conducted in order to elucidate how mechanical signals are transferred into bone cellular activity. This scarcity is largely due to the lack of viable options of tools that can be used in in vivo studies to incur oscillatory fluid flow inside the bone intramedullary cavity. A few in vivo studies have been performed with bulky external oscillatory loading equipment (e.g., microfluidic syringe pumps) [22,23,24]. However, syringe pumps placed outside of the body require wired catheters surgically inserted through the skin to the bones of test subjects. Such methodologies impose strong restrictions on the normal physical activities of test subjects, which greatly limits the potential for more rigid in vivo studies of investigating bone adaptation to bone intramedullary fluid pressure/flow modulation without mechanical loading. Alternative tools with minimal invasiveness and minimal restrictions on physical activities of test subjects are highly desirable.

In this work, we report a magnetically operated, battery-less, and implantable microfluidic pump that is designed for in vivo studies of bone intramedullary fluid modulation for the potential application in bone density augmentation study in rat femora. 

## 2. Device Design, Fabrication, and Experimental Setup

### 2.1. Design Criteria and Working Principles

**Figure 1:**
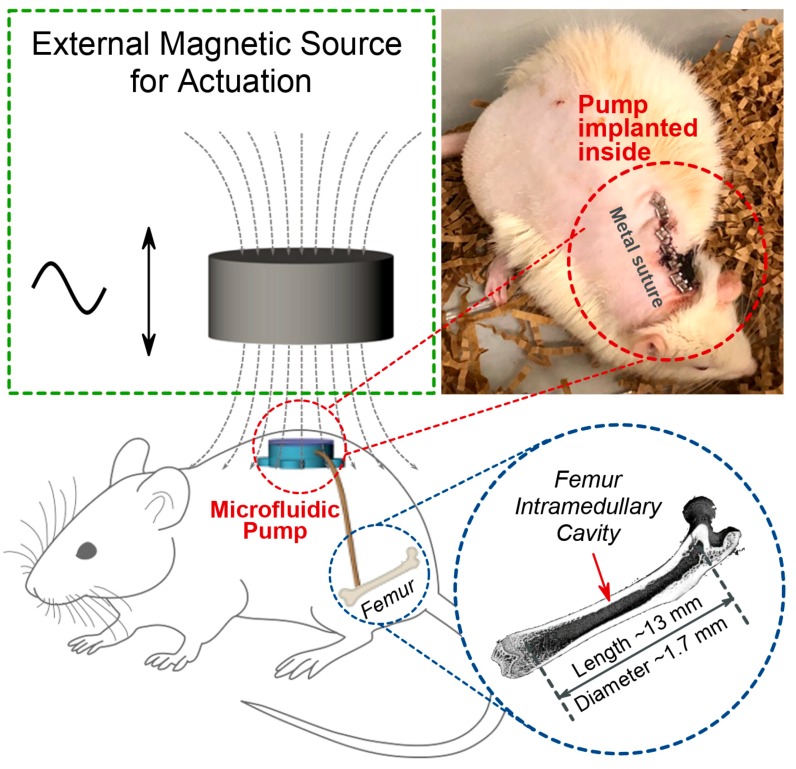
Working principles of the wireless, battery-less, implantable, magnetically operated microfluidic pump for modulation of fluid flow in the intramedullary cavity of rat femora. A photomicrograph shows the microfluidic pump implanted underneath the skin at the back of a rat.

There are a few criteria that must be considered for the design of an implantable microfluidic pump for in vivo study. First, we envisioned using the femora of Fischer-344 rats [25] for in vivo study. The size of the targeted bone intramedullary cavity in a six-month-old Fisher-344 rat is approximately 13 mm in length and 1.7 mm in diameter (Figure 1). This determines the outer diameter of the tubing which connects to the bone intramedullary cavity, and the implanted microfluidic pump must be smaller than 1.7 mm. Second, the microfluidic pump must be as small as possible to be implanted in rats. Because of available sub-dermal volume and convenience of wireless remote operation, it was determined that we would place the magnetic microfluidic pump in the back of the rats. Since the microfluidic pump is operated magnetically, the pressure variation created by the microfluidic pump depends on the size of magnets. We studied different sizes of permanent magnets in millimeter ranges in the implantable microfluidic pump. Third, any device that would be implanted in the body must be biocompatible and thoroughly sterilized. We decided to use polydimethylsiloxane (PDMS) as the material for the body of the implantable microfluidic pump because of its proven biocompatibility [26] and ease of fabrication. 

Figure 1 shows the schematic diagrams for the working principle of the wirelessly operated implantable magnetic microfluidic pump for the intramedullary cavity fluid modulation of animal. A small magnetically operated microfluidic pump is implanted underneath the skin at the back of a Fisher-344 rat. The implanted magnetic microfluidic pump is connected into the intramedullary cavity in the femur of the Fischer-344 rat through a tube. The implanted magnetic microfluidic pump is operated by reciprocating linear actuation of an external magnet. A photomicrograph shows surgically implanted microfluidic pump in the back of a six-month-old Fischer-344 rat. 

### 2.2. Fabrication of the Implantable Microfluidic Pump

**Figure 2:**
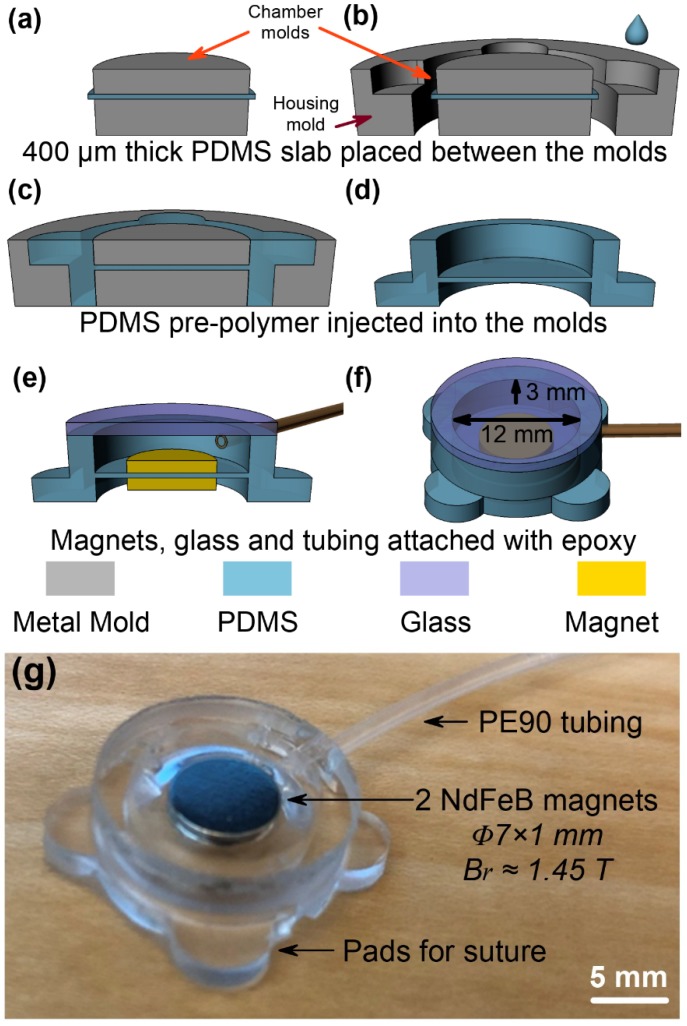
Schematic diagrams of the fabrication process: (**a**) 400 μm thick polydimethylsiloxane (PDMS) membrane clamped between two pieces of stainless-steel cylindrical chamber mold, (**b**) chamber mold and PDMS membrane placed into the center of housing mold and injected with PDMS pre-polymer, (**c**) PDMS curing in oven, (**d**) cured PDMS peeled off from the molds, (**e**) borosilicate glass, NdFeB magnets, and PE90 tubing attached, (**f**) schematic in perspective views and (**g**) optical image of the fabricated magnetic microfluidic pump with two NdFeB magnets (N50 grade) of size Φ7 × 1 mm.

The implantable microfluidic pump was fabricated by replication of PDMS from a mold. Figure 2a–f shows the sequence of the fabrication process. A set of stainless-steel molds were manufactured in a machine shop, and the molds were cleaned by acetone, isopropyl alcohol (IPA), and de-ionized (DI) water prior to use. The pre-polymer of PDMS was prepared by manually mixing two-part silicone (Sylgard 184, Dow Corning, Midland, MI, USA) in 10:1 wt%, followed by degassing in a vacuum chamber for 2 h. Approximately 400 μm thick PDMS membrane was obtained by casting in a glass container. The 400 μm PDMS membrane was then cut and clamped between two cylindrical stainless-steel plates (12 mm in diameter; 3 mm and 2 mm in thickness, respectively) in the chamber mold (Figure 2a). The chamber mold with the clamped 400 μm PDMS membrane was placed into the center of the housing mold (Figure 2b). Liquid PDMS pre-polymer was slowly injected into the gaps of the housing mold by using a syringe, and then baked in an oven at 90 °C for 30 min to cure the PDMS (Figure 2c). The replicated PDMS structure was carefully peeled off from the chamber and housing molds (Figure 2d). Two identical NdFeB permanent magnets (N50 grade, B_r_~1.45 T) of various sizes (4 mm~8 mm in diameter and 0.5 mm~1 mm in thickness) were attached to the top and bottom sides of the 400 μm thick PDMS membrane (Figure 2e). A pre-cut circular borosilicate glass of 1 mm in thickness and PE90 tube (ID: 0.86 mm, OD: 1.27 mm, BD 427421, Beckton Dickson, Franklin Lakes, NJ, USA) were bonded using medical grade epoxy (M-31CL, Henkel Loctite, Düsseldorf, Germany). The size of the microfluidic pump chamber was 12 mm in diameter and 3 mm in depth (Figure 2f). The outer diameter of the microfluidic pump chamber was 16 mm and four pads were designed for easier suture which made the total external dimension of the microfluidic pump 22 mm in diameter. Figure 2g shows a photomicrograph of the fabricated magnetic microfluidic pump with NdFeB magnets (7 mm diameter 1 mm in thickness). 

### 2.3. Experimental Setup

#### 2.3.1. External Magnetic Actuator

The external magnetic force was provided by a reciprocally moving external NdFeB permanent magnet (N50 grade, B_r_~1.45 T). The reciprocal movement of the external magnet was achieved by attaching the magnet to a linear reciprocating actuator with a maximum speed of 160 rpm and stroke distance of approximately 70 mm. The microfluidic actuator remains relatively still while actuated, the change of relative distance between the external and internal magnets modulates the magnetic field and its gradient on the magnets embedded in the microfluidic pump. Such modulation leads to a sinusoidally modulated magnetic force exerted onto the magnets attached on the membrane of the microfluidic pump. 

#### 2.3.2. Static and Dynamic Pressure Measurements

Static pressure measurements were carried out using a microfluidic pump with two identical internal magnets of various sizes (4–8 mm in diameter, 0.5–1 mm in thickness). These microfluidic pumps were connected to a commercial pressure sensor. The distance between the bottom surface of the external magnet and the top surface of the microfluidic pump was set at 15 mm and 85 mm. The distance between the external and the internal magnets were 18 mm and 88 mm. The induced pressure elevation was subtracted by static pressure.

Dynamic pressure measurements were carried out using a microfluidic pump with two identically sized internal magnets, 7 mm in diameter and 1 mm in thickness. The distance between the bottom surface of the external magnet and the top surface of the microfluidic pump was controlled in the range of 15–85 mm (distance between magnets 18–88 mm). The microfluidic chamber and the tube were filled with an incompressible fluid, 0.9% heparinized saline. Dynamic pressure was measured by a commercial pressure sensor using PE90 tubes 25 cm, 50 cm, and 75 cm in length.

#### 2.3.3. In Vivo Dynamic Experiments

In vivo dynamic experiments were performed with a microfluidic pump described in ex vivo dynamic experiments with a 6-month-old Fischer-344 rat. The distance between the bottom surface of the external magnet and the top surface of the microfluidic pump was controlled in the range of 15–85 mm (distance between magnets 18–88 mm). The microfluidic pump and the intramedullary cavity of the rat’s femur were connected using PE90 tubes 25 cm, 50 cm, and 75 cm in length. Due to the size limitation of the femur and intramedullary cavity, the PE90 tube was connected with a 10 mm long PE50 tube (ID: 0.58 mm, OD: 0.97mm, BD 427411) which was inserted into the intramedullary cavity of the rat’s femur. The microfluidic pump chamber and the tubes were filled with 0.9% heparinized saline which is incompressible and prevents blood clotting [27,28]. 

## 3. Results

### 3.1. Static Ex Vivo Pressure Measurements

Static ex vivo tests were first conducted to study the relationship of induced pressure elevation with respect to static magnetic force. The axial magnetic force exerted on the internal magnets by external magnetic field gradient can be estimated by [29]

(1) $$F_{z}\approx \frac{1}{\mu_{0}}B_{r}V_{m}\frac{dB_{z}}{dz}$$

where $F_{z}$ is the axial magnetic force caused by external magnetic field gradient, $\mu_{0}$ is the permeability of vacuum ($12.57\;\times 10^{-7}\;H/m$), $B_{r}$ is remnant magnetic flux density of the internal magnets in the microfluidic pump (~1.45 T), $V_{m}$ is the total volume of the two internal magnets which can be calculated as $V_{m}=2\pi r^{2}h$, with r and h denoting the radius and height of the internal magnets, and $dB_{z}/dz$ is the external magnetic flux density gradient along the axial direction of the external magnets. The pressure elevated by external magnetic force is given by [30]

(2) $$P=\frac{F_{z}}{A}$$

where P is the pressure excited by magnetic force, $F_{z}$ is the axial magnetic force exerted on the internal magnets by external magnetic field gradient, and A is the area of the diaphragm which was calculated as $A=\pi r^{2}$ ($1.13\;\times \;10^{-4}\;m^{2}$). 

**Figure 3:**
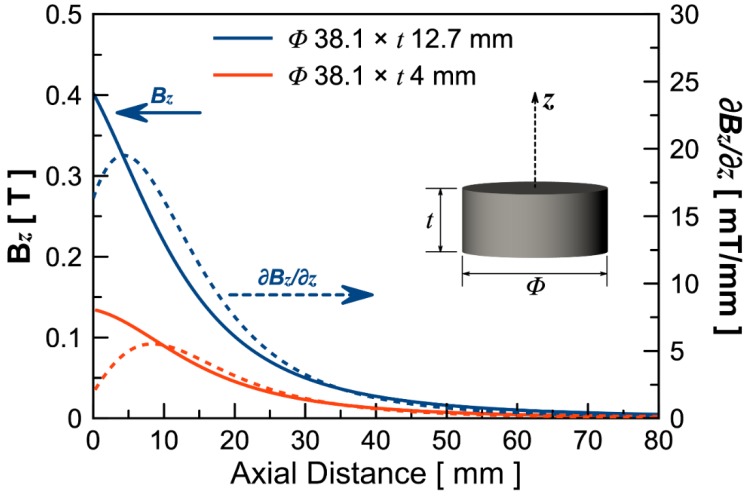
Simulation results of magnetic flux density (solid lines) and magnetic flux density gradient (dotted lines) generated by the external magnet along the axial direction. Blue lines are results for the larger magnet (38.1 mm in diameter and 12.7 mm in thickness) and red lines are results for the smaller magnet (38.1 mm in diameter and 4 mm in thickness).

Finite element method (FEM) by COMSOL Multiphysics^®^ was utilized to study the magnetic field of the external magnets. Figure 3 shows the theoretical results of magnetic flux density and the magnetic flux density gradient of two cylindrical external magnets with diameters of 38.1 mm and varied thicknesses of 12.7 mm and 4 mm. As expected, both the magnetic flux density and flux density gradient of the larger magnet is greater than those of the smaller magnet. 

**Figure 4:**
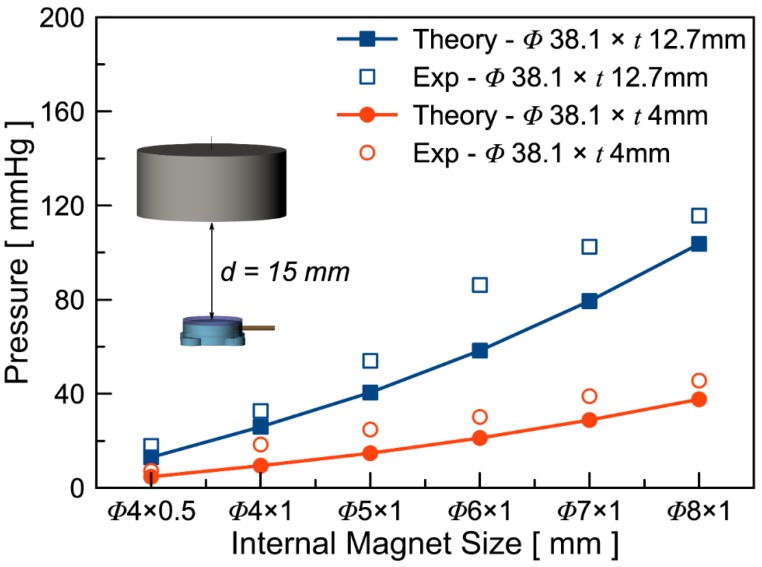
Theoretical and experimental results of the static pressure of various internal magnet sizes under an external magnetic field caused by two different external magnets. The distance between the bottom of the magnet and the top of the microfluidic pump is controlled at 15 mm.

According to Equations (1) and (2), larger gradient and larger volume of the internal magnets should lead to higher magnetic force and hence a larger pressure variation. A set of devices with various internal magnet sizes were fabricated to experimentally validate the theoretical model. The experiments were conducted by connecting the pump directly to a commercial pressure sensor. The distance between the bottom surface of the external magnet and the top surface of the microfluidic pump was set as 15 mm and 85 mm. The distance between the external and the internal magnets were 18 mm and 88 mm. Figure 4 shows the theoretical and experimental results of the static pressure induced by external magnetic force with the inset showing the experimental setup. As shown in the figure, the overall static pressure elevation is higher with the thicker external magnets regardless of the sizes of the internal magnets. This is in accordance with the higher magnetic field gradient generated by the thicker magnet. Furthermore, the static pressure elevation increases as the size of the internal magnets increases. This is expected as the magnetic force is proportional to the volume of the internal magnet as shown in Equation (1). It is also worth noting that the experimental results are higher than the theoretical values. This is likely due to fringing field effect caused by slight axial misalignment (e.g., off from the collinear axis) and angular misalignment. 

### 3.2. Dynamic Ex Vivo Pressure Measurements

Ex vivo dynamic pressure measurements of the microfluidic pump were carried out to test the output performance of the device. The pressure measurement was conducted with fluid modulator filled with 0.9% heparinized saline, and an approximately 40 cm long PE90 tube was connected to a commercially available pressure sensor (SP844, MEMSCAP, Durham, NC, USA) to monitor real-time pressure modulation. 

**Figure 5:**
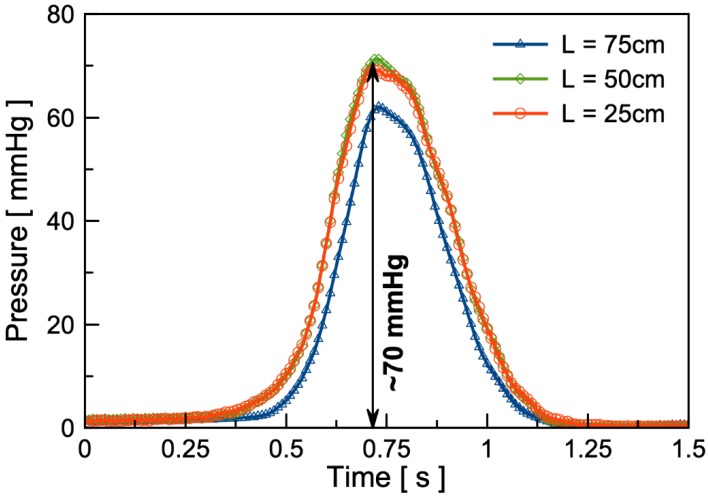
Ex vivo dynamic pressure response of the microfluidic pump connected to PE90 tubes 75 cm, 50 cm, and 25 cm in length using 0.9% heparinized saline. The external magnet used is a NdFeB magnet with a diameter of 38.1 mm and 12.7 mm thick operated at distance ranging from 15 mm to 85 mm.

As shown in Figure 5, the results show that the pressure modulation amplitude reaches approximately 70 mmHg under the external magnetic field with a separation distance ranging from 15 mm to 85 mm (~120 mT amplitude) between the external magnet and the internal magnet inside the microfluidic pump. Due to a low excitation frequency (~0.5 Hz) and low volumetric flow rate, the pressure drops in various tube lengths are relatively small.

### 3.3. In Vivo Intramedullary Pressure Modulations

The in vivo study of the microfluidic pump was also conducted to test the induction of on-demand modulation of bone intramedullary fluid flow and pressure. The experimental pressure measurements were carried out on a 6-month-old Fischer-344 rat, with a reciprocating external magnet (diameter of 38.1 mm and 12.7 mm thick) as an excitation source. The separation distance is, again, controlled, ranging from 18 mm to 88 mm to modulate the external magnetic field gradient.

**Figure 6:**
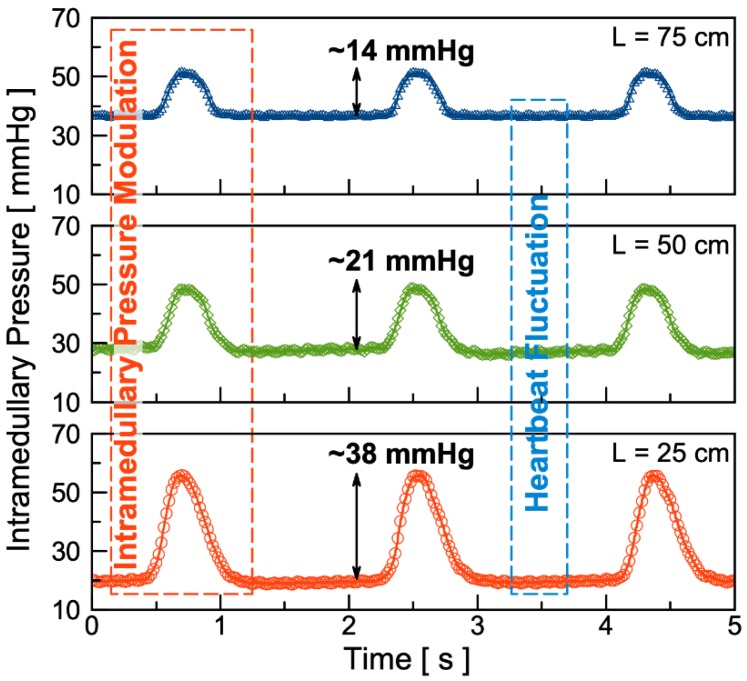
Test results of in vivo intramedullary fluid modulation on a six-month-old Fischer-344 rat using PE90 tubes 75 cm (top), 50 cm (middle), and 25 cm (bottom) in length. The external magnet used is a NdFeB magnet with a diameter of 38.1 mm and 12.7 mm thick, operated at distance ranging from 18 mm to 88 mm.

Figure 6 shows the in vivo experimental results of the fluid pressure modulation under ~0.5 Hz of external excitation inside of bone intramedullary cavity. The intramedullary pressure modulation amplitude with 25 cm, 50 cm, and 75 cm long PE90 tubes are found to be 38 mmHg, 21 mmHg, and 14 mmHg, respectively. In comparison, a minor pressure fluctuation of less than 1 mmHg amplitude was also observed. Such a minor pressure fluctuation is likely attributed to heartbeat of the rat, approximately 400 beats/min [31]. Compared to relatively consistent pressure modulation amplitude of approximately 70 mmHg, the in vivo pressure measurement results show that as the length of the tube increases, the pressure modulation amplitude decreases. This pressure drop and dependence of tube length could be partially attributed to contents of the porous structure inside the bone intramedullary cavity, structured by densely distributed blood, nerve fibers, and mesenchymal stem and stromal cells. 

## 4. Conclusions

We report a wireless, battery-less, and implantable magnetically-operated intramedullary microfluidic pump. Ex vivo experimental results of static and dynamic pressure modulation are presented to show the effectiveness of the fabricated microfluidic pump to induce on-demand pressure modulation. The results show that the microfluidic pump induces ~102 mmHg static pressure elevation and ~70 mmHg dynamic pressure modulation amplitude under external magnetic excitations. The in vivo test was conducted with a Fischer-344 rat to confirm its implantability and capability to induce on-demand dynamic intramedullary fluid pressure modulations. The results show that the pressure modulation amplitude with 25 cm, 50 cm, and 75 cm long PE90 tubes are 38 mmHg, 21 mmHg, and 14 mmHg, respectively. These results confirm that the wireless and implantable microfluidic pump is fully functional. This study can be extended for use in in vivo studies of bone density augmentation and potential osteoporosis treatment.
